# Methylation Markers of Early-Stage Non-Small Cell Lung Cancer

**DOI:** 10.1371/journal.pone.0039813

**Published:** 2012-06-29

**Authors:** Kaie Lokk, Tõnu Vooder, Raivo Kolde, Kristjan Välk, Urmo Võsa, Retlav Roosipuu, Lili Milani, Krista Fischer, Marina Koltsina, Egon Urgard, Tarmo Annilo, Andres Metspalu, Neeme Tõnisson

**Affiliations:** 1 Institute of Molecular and Cell Biology, University of Tartu, Tartu, Estonia; 2 Department of Genetics, United Laboratories, Tartu University Hospital, Tartu, Estonia; 3 Lung Clinic, Tartu University Hospital, Tartu, Estonia; 4 Institute of Computer Science, University of Tartu, Tartu, Estonia; 5 Department of Pathology, Tartu University Hospital, Tartu, Estonia; 6 Estonian Genome Center, University of Tartu, Tartu, Estonia; University of Barcelona, Spain

## Abstract

**Background:**

Despite of intense research in early cancer detection, there is a lack of biomarkers for the reliable detection of malignant tumors, including non-small cell lung cancer (NSCLC). DNA methylation changes are common and relatively stable in various types of cancers, and may be used as diagnostic or prognostic biomarkers.

**Methods:**

We performed DNA methylation profiling of samples from 48 patients with stage I NSCLC and 18 matching cancer-free lung samples using microarrays that cover the promoter regions of more than 14,500 genes. We correlated DNA methylation changes with gene expression levels and performed survival analysis.

**Results:**

We observed hypermethylation of 496 CpGs in 379 genes and hypomethylation of 373 CpGs in 335 genes in NSCLC. Compared to adenocarcinoma samples, squamous cell carcinoma samples had 263 CpGs in 223 hypermethylated genes and 513 CpGs in 436 hypomethylated genes. 378 of 869 (43.5%) CpG sites discriminating the NSCLC and control samples showed an inverse correlation between CpG site methylation and gene expression levels. As a result of a survival analysis, we found 10 CpGs in 10 genes, in which the methylation level differs in different survival groups.

**Conclusions:**

We have identified a set of genes with altered methylation in NSCLC and found that a minority of them showed an inverse correlation with gene expression levels. We also found a set of genes that associated with the survival of the patients. These newly-identified marker candidates for the molecular screening of NSCLC will need further analysis in order to determine their clinical utility.

## Introduction

Lung cancer is the leading cause of cancer-related deaths in the world. Epigenetic events are early and frequent in carcinogenesis [1], [2], [3], which makes DNA methylation an attractive biomarker for cancer. Epigenetic events could also provide a tractable link between the genome and the environment, with the epigenome serving as a biochemical record of relevant life events, e.g. cigarette smoking [4].

Lung cancer is morphologically divided into non-small cell and small cell lung cancer (NSCLC and SCLC). NSCLC accounts for about 80% of the lung cancers and is a heterogeneous clinical entity with major histological subtypes such as squamous cell carcinoma (SCC), adenocarcinoma (AC) and large cell carcinoma [5]. A common feature of the different subtypes of NSCLC is the somewhat slower growth and spread compared to SCLC, enabling surgical eradication in its early stages. Only a minor fraction of NSCLC cases are currently diagnosed in clinical stages I to IIb, where surgical removal is the therapy of choice.

The biomarker-driven approach at preinvasive phases could aid in diagnosing or ruling out lung cancer. Current markers, including squamous cell carcinoma antigen, carcinoembryonic antigen, cytokeratin 19 fragment antigen 21-1 and neuron-specific enolase were shown to lack satisfactory diagnostic power. In a recent study, only 37.3% of early-stage lung cancers could be diagnosed using the combination assays of the above-mentioned tumor markers [6].

Our study was aimed at the genome-wide identification of DNA methylation-based biomarker candidates in early-stage NSCLC. DNA methylation occurs vastly in the context of cytosine-guanine dinucleotides (CpGs) [7]. CpG-rich short stretches (CpG islands) are usually located in the promoter region of genes and are normally kept in the demethylated state [8]. In cancer, CpG islands located in the promoter area of tumor suppressor genes and “house-keeping” genes become hypermethylated, which can lead to decreased expression of these genes. At the same time, the genome is globally demethylated, which in turn can lead to the activation of oncogenes [9], [10]. Methylation of CpG island shores – regions with lower CpG density within approximately 2 kb of CpG islands - is also closely associated with transcriptional inactivation [11].

Recently, significant progress has been made in the genome-wide DNA methylation analysis. The methods include bisulfite conversion of DNA, immunoprecipitation or affinity purification of methylated DNA followed by microarray analysis or high-throughput sequencing [12]. It has been shown that in terms of accuracy, bisulfite-based methods perform slightly better than enrichment-based methods and do not require a statistical correction for CpG bias [13].

We have performed a genome-wide DNA methylation study of stage I NSCLC to obtain an insight into early-stage epigenetic alterations in lung cancer and identify potential diagnostic or prognostic biomarkers. In our study we used the HumanMethylation27 BeadChips (Illumina, Inc) that enable cost-effective quantitative comparisons across many samples.

## Results

### CpG Methylation Analysis

**Figure 1:**
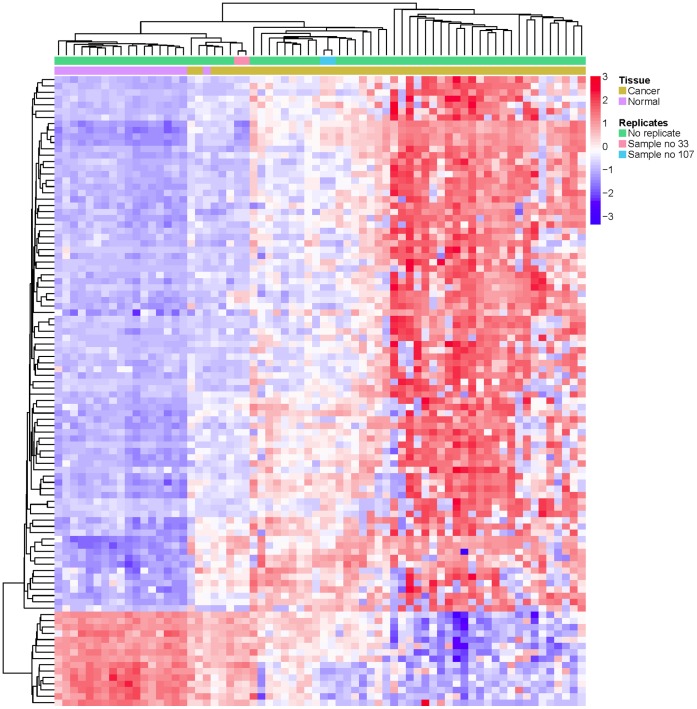
Differential DNA methylation between NSCLC and normal lung samples. DNA methylation levels are shown for the top 100 CpG sites with the highest delta Beta values (FDR corrected) of DNA methylation between cancer tissue and normal lung tissue. Methylation Beta-values are represented as row Z-scores. A heatmap was generated using unsupervised 2D hierarchical cluster analysis. Red indicates high methylation and blue indicates low methylation relative to the row mean.

Overall, we detected 496 CpGs in 379 genes hypermethylated and 373 CpGs in 336 genes hypomethylated in NSCLC (Figure S1, Table S1). A heatmap of the 100 most differently methylated genes in NSCLC compared to control samples is shown in Figure 1.

Compared to AC samples, SCC samples had 263 CpGs in 223 hypermethylated genes and 513 CpGs in 436 hypomethylated genes (Figure S2, Table S2).

Two DNA samples (IDs 33 and 107) were processed in duplicate as an internal control of the assay’s reproducibility. Pearson’s correlation coefficient for both duplicates was 0.998. Methylation levels determined by the Infinium assay were validated using Sanger sequencing of bisulfite-converted DNA for 11 CpGs (six CpGs from NSCLC versus control samples analysis and five CpGs from survival analysis). The mean correlation between two methods was 83% (range 48.9%; 97.4%) (Figure S3).

**Figure 2:**
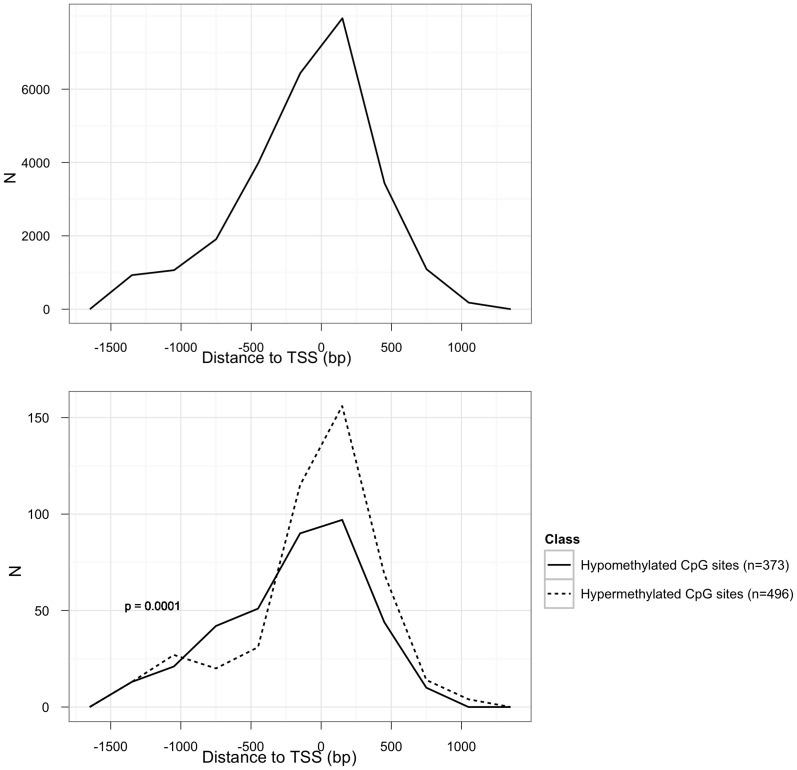
CpGs’ distance from TSS. We measured CpGs’ distance from the transcription start site (TSS). a) Distance from TSS of all the CpGs on the methylation array. b) Distance from TSS of hypermethylated CpGs (dotted line) and distance from TSS of hypomethylated CpGs (continuous line). On the x-axis, the distance from TSS is measured in bp-s, and on the y-axis N represents the number of CpGs.

The CpG sites analyzed by HumanMethylation27 assay are located within 1.5 kb upstream to 1 kb downstream of the TSS of their respective genes. We found a significant difference in the location of hypermethylated vs. hypomethylated CpGs relative to the closest TSS (p = 0.0001, Welch Two Sample t-test). Hypermethylated CpGs were preferentially located at TSSs, 3′ downstream of the TSS or in CpG islands. In contrast, hypomethylated CpGs were frequently found 5′ upstream of the respective TSSs or in CpG island shores [11] (Table 1, Figure 2).

**Table 1:** Distance of differentially methylated CpGs to the nearby transcription start sites (TSS, FDR corrected p<0.05, mean difference in methylation level in NSCLC vs tumor-free lung at least 13.6%).

	Hypermethylated CpGs	Hypomethylated CpGs
Distance to TSS (median; 1^st^ quartile; 3^rd^ quartile)	**20.0**; −215; 229	**−63**; −469.2; 132.5
Located inside CpG island	86% (429)	23% (86)
Located outside CpG island	14% (67)	77% (287)

Distance to TSS of hyper- vs hypomethylated CpGs differed by p = 0.0001 (Welch Two Sample t-test).

Different CpGs of *HSPA12B*, *PABPC5* and *TP73* were either hyper- or hypomethylated in NSCLC. In the *TP73* gene, the hypermethylated CpG site in the tumor sample was located upstream of the TSS of the full-length mRNA isoform. Hypomethylated *TP73* CpGs were located close to the TSSs of the shorter isoforms. Hypermethylated CpGs of the *HSPA12B* and *PABPC5* were located in their 5′ CpG islands, whereas hypomethylated CpGs were located upstream, outside the CpG islands.

### Gene Expression Microarray Validation by qRT-PCR

**Figure 3:**
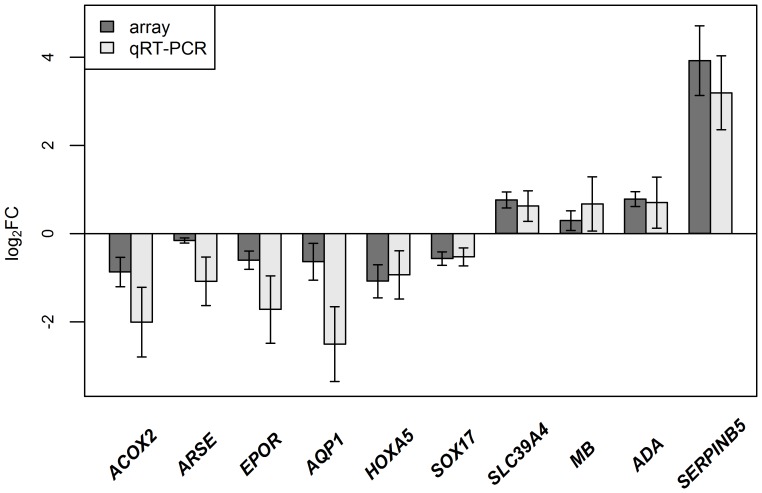
The concordance between microarray and qRT-PCR measurements. On the y-axis is shown average log2fold-change determined by Illumina array and qRT-PCR (8 sample pairs). Error bars indicate standard error of the mean (SEM).

Gene expression array setup and results are reported in the recent paper [14]. 10 genes and eight sample pairs were used to validate microarray results, using qRT-PCR technology. All the genes showed the same direction of over- or underexpression in the lung cancer samples, using both technologies (Figure 3). Seven genes showed a significant correlation between the expression fold-changes determined by qRT-PCR and microarray (p<0.05, R >0.7) (Figure S4).

### Methylation related to Gene Expression Changes

Using Pearson’s correlation analysis, we were able to determine the expected inverse correlation between the differential methylation levels and gene expression values for 378 (43.5%) of the 869 differentially methylated CpGs between NSCLC and control samples. In different histological groups we were able to find 337 of 780 CpGs (43.2%), the methylation levels of which were inversely correlated to the gene expression levels.

We performed a qPCR analysis of the different *TP73* isoforms to test whether differential methylation next to the TSSs of different isoforms affects their expression level. The qPCR analysis did not reveal a statistically significant difference (p-value 0.36, paired t-test) between the expression levels of the two isoforms in our tumor samples.

### Ingenuity Pathway Analysis

We performed *in silico* functional and interaction analyses of the differentially methylated genes using Ingenuity Pathway Analysis (IPA) software (Ingenuity Systems, Redwood City, CA), and found 78 network eligible genes and 451 Functions/Pathways eligible genes. By including the known direct and indirect interactions, the most prominently represented gene network was related to tumor necrosis factor (TNF, Figure S5). Most of the genes (n = 22) in the network were hypomethylated, but some genes (n = 7) were also hypermethylated.

### DNA Methylation related to Smoking Behavior

Based on tobacco smoking pack-years data, we asked whether smoking affects the DNA methylation patterns in a tumor. One patient who lacked smoking data was excluded. Linear regression analysis was performed using the Bioconductor Limma package. Analysis within tumor samples did not show any differentially methylated genes related to the extent of tobacco smoking. Comparing the limited number of non-smokers (n = 3, 6.4%) with smokers (n = 44, 93.6%) we found four differentially methylated CpG-sites in three genes (p<0.05, FDR adjusted), which are all hypomethylated in smokers group: *CXorf38*, *MTHFD2* and *TLL2*.

### Altered Methylation and Long-term Survival

We performed two types of survival analyses to find potential prognostic methylation markers. The patients with only up to one month survival after surgery (n = 2) were excluded to avoid any potential confounding influence of postoperative complications.

**Figure 4:**
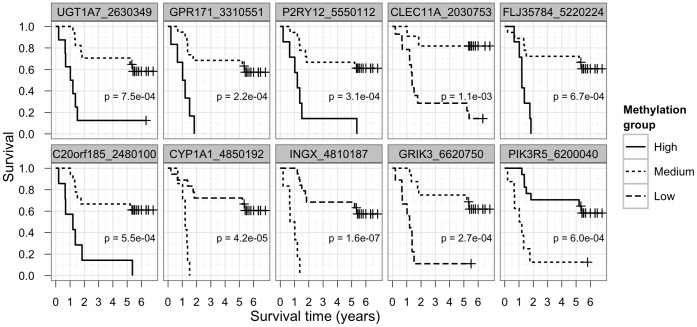
Survival curves of 10 differentially methylated CpG sites. We performed a survival test on each of the CpG sites. The methylation values are divided into 3 groups: low (0–0.25), medium (0.25–0.75) and high (0.75–1). As a result we found 10 CpG sites whose methylation level differs in different survival groups. The x-axis shows survival in years and the y-axis shows overall survival.

Firstly, we performed a Kaplan-Meier survival analysis on each of the CpG sites by dividing the Beta values into low, medium and high methylation groups. We only report results where all groups were larger than five patients. As a result, we found 10 CpGs in 10 genes, with methylation level differences in different survival groups (Figure 4). Patients with a medium methylation level of *UGT1A7, GPR171, P2RY12, FLJ35784* and *C20orf185* had better survival than patients with high-level methylation. Patients with a medium methylation level of *CLEC11A* and *GRIK3* had better survival compared to low-level methylation. Patients with a low methylation level of *CYP1A1* and *INGX* had better survival than those with medium-level methylation. Patients with a high methylation level for *PIK3R5* had better survival than those with medium-level methylation.

Secondly, we performed the differential methylation analysis by combining Cox proportional hazard analysis and Wilcoxon rank-sum test. We found 18 CpGs in 15 genes in patients with 1 to 24 months survival (n = 12) vs. patients with 60 months and longer survival (n = 15), p<0.05, and the methylation difference cut-off applied. From the differentially methylated genes, *DXS9879E* (*LAGE3*), *RTEL1* and *MTM1* were hypermethylated, and *SCUBE3*, *SYT2*, *KCNC3*, *KCNC4*, *GRIK3*, *CRB1*, *SOCS2*, *ACTA1*, *ZNF660*, *MDFI*, *ALDH1A3* and *SRD5A2* were hypomethylated in the group with poor survival (Figure S6).

## Discussion

We have performed a genome-wide DNA methylation study in 48 stage I NSCLC patients and 18 macroscopically cancer-free control samples by using cluster analysis to search for genes that distinguish between the cancerous and normal lung tissue and compared these genes’ methylation levels with their expression levels. In addition, we performed *in silico* functional and interaction analysis of differently methylated genes using IPA software. Linear regression was used to find genes related to smoking, and Kaplan-Meier survival analysis was performed to identify the differential methylation of genes related to patient survival.

As a result, we detected 496 CpGs in 379 hypermethylated genes and 373 CpGs in 336 genes that were hypomethylated in NSCLC. A perfect separation of the control lung tissue samples from NSCLC samples was not obtained, as one normal lung sample clustered together with the cancer samples and six cancer samples (one in replicate) showed methylation patterns somewhat resembling the tumor-free lung tissue. Since we used non-dissected tumor samples, this finding may be at least partially caused by the confounding effect of non-neoplastic tissue present in these samples. Pathological examination of the NSCLC samples with DNA methylation profiles similar to the normal lung samples revealed either low tumor content (10 to 30%) or a very heterogeneous composition of tumor cells in the samples. These co-clustering cancer samples and cancer-free lung samples were therefore excluded from further analyses.

Our first goal was to identify the genes involved in early events in tumor-specific methylation. As a result, our screening uncovered some well-known methylation markers, some of which have tumor suppressor activity: *CDKN2A*, *MGMT*, *GATA4*, *HOXA7*, *HOXA9*, *RUNX3*, *SFRP1*. Most of the identified genes are novel methylation markers for NSCLC, although some of these have been described as methylated in other cancers. *LGALS12* is a candidate tumor suppressor that is able to arrest the cell cycle and inhibit the proliferation of several cancer cell lines [15]. In breast cancer, *LGALS12* has been found downregulated in the malignant tissue [16].

The second goal of our study was to correlate the methylation level changes with the gene expression data. Using Pearson’s analysis, we were able to find the expected inverse correlation between the methylation levels and gene expression values for 378 of 869 CpG sites (43.5%). The highest inverse correlation values between hypermethylated CpGs and expression values were found for the *AGER* (−0.78), *EPOR* (−0.65) and *AQP1* (−0.63) genes. The mean distance of TSS of inversely correlated genes was −65 bp (range −1498; 917 bp) compared to −158 bp (range -1474; 818 bp) of positively correlated CpGs (p-value 0.02, students t-test). The range of CpGs is probably affected by the fact that Illumina’s Infinium HumanMethylation27 microarray covers CpGs that are located predominantly in the vicinity of promoter regions and not in the gene body or 3′UTR. Aquaporin 1 (*AQP1*) has been reported to be hypermethylated and downregulated in NSCLC [17]. *AGER*, which encodes a receptor of the immunoglobulin superfamily, has been reported to be downregulated in NSCLC [18]. The highest inverse correlation values for hypomethylated CpG sites and expression values were found for *MB* (−0.63), *ADA* (−0.60) and *MAGEA6* (−0.60) genes. Myoglobin (*MB*) is associated with tumor progression and helps to overcome hypoxia in cancer cells [19]. Hypermethylation and/or the downregulation of some of the genes identified in our study (e.g. *ALDH1A2*, *HOXA5*, *MT1E*, *SOX17*) have been reported in other cancers [20], [21], [22], [23], but not yet in lung cancer. It is hypothesised that *RASSF1* acts as a tumor suppressor in lung cancer progression [24]. Among the hypomethylated and upregulated genes, the most frequently reported gene was *SERPINB5* (Maspin). Overexpression of *SERPINB5* has been associated with cancer progression and a poor prognosis in lung cancer [25].

We can assume that genes with an inverse correlation have a higher likelihood of being regulated by methylation. However, many genes probably become methylated randomly during carcinogenesis, and this does not necessarily have a steady state effect on gene expression levels.

In the case of different histology groups, we were able to find an inverse correlation between the differentially methylated CpG sites and gene expression values for 337 of 780 CpG sites (43.2%). The highest inverse correlation values were for *ACOX2* (−0.75), *ARSE* (−0.70) and *SLC39A4* (−0.68), which have not been associated with NSCLC histological subtypes before. Of the inversely correlated genes, it has been reported that *PIGR*, *MUC1* and *FOLR1* are downregulated in SCC compared to AC [26], [27], [28], which is consistent with our results.

Analysis using IPA software revealed that the dominant functions of the differentially methylated genes were cell-to-cell signaling and interaction, DNA replication and repair, cellular growth and proliferation, cell death, cancer, inflammatory response, etc. A number of gene functions, such as cell growth, proliferation and cell death, are directly involved in cancer progression, but the immune system also plays an important role in fighting cancerous cells. It is also widely known that deficiencies in pathways of DNA repair and damage control are responsible for most or even all human cancers. After including the known indirect relations, the most prominent gene network revealed was related to *TNF* (Figure S5). *TNF* has a dual role in tumor biology. It is a cytokine with well-known anticancer properties, but may also promote cancer development and progression [29]. Hypomethylated genes in the *TNF* network were cytokines (*CCL3, CCL4, CCL7, CCL8, CCL22, IL21, IL17A, EBI3*) that can either stimulate or inhibit tumor growth and progression. The *TNF* network also includes the well-known potent antiapoptotic gene *BCL2*, which was also mostly hypomethylated in our NSCLC samples. *ALDH1A3* (aldehyde dehydrogenase 1 family, member A3) indirectly regulated by *TNF* plays a role in the detoxification of aldehydes generated by alcohol metabolism and lipid peroxidation. Hypomethylation of this gene was related to a worse prognosis in our study cohort (Figure S6).

We found that *TP73* had three differentially methylated CpGs, two of which were hypomethylated in the tumor tissues and located near the TSS of its shorter isoforms. The hypermethylated CpG was located upstream of the TSS of the full-length mRNA isoform. We measured the expression of different isoforms using qPCR analysis, but did not find any statistically significant differences (p-value 0.36, paired t-test) in the tumor samples, although the long isoform was expressed at a slightly lower level than the short isoform. We performed a Kaplan-Meier survival test for CpG sites, for which we divided their methylation values into 3 groups: low (0–0.25), medium (0.25–0.75) and high (0.75–1). As a result, we found 10 CpGs in 10 genes whose methylation level differs in different survival groups (Figure 4). *UGT1A7* is an enzyme involved in the metabolism of (pre)carcinogens present in tobacco smoke. Precarcinogens and their metabolites are considered to play an important role in the carcinogenesis of the tobacco smoke-related cancers [30]. It has been shown that polymorphisms in the UGT1A7 gene are associated with lung cancer, suggesting that these polymorphisms reduce enzymatic activity [31]. A high methylation level could also affect *UGT1A7* activity and cause a poor prognosis for NSCLC patients. *CYP1A1* (belonging to cytochrome P450 superfamily) catalyzes many reactions involved in drug metabolism, also the conversion of polycyclic aromatic hydrocarbons into reactive metabolites and detoxifications of environmental carcinogens. It has been shown that *CYP1A1* is hypermethylated in lung cancer samples compared to normal lung samples, and this was associated with reduced mRNA levels [32]. In our study, higher methylation levels were associated with poor survival of lung cancer patients that supports the hypothesis that *CYP1A1* may have protective role in cancer progression. Other genes found in survival analysis have not been reporter to be involved in lung cancer patients’ survival.

Using a different type of survival analysis, where the differential methylation analysis was performed by combining Cox proportional hazard analysis and the Wilcoxon rank-sum test, we were able to analyze 12 patients with a survival of less than 24 months and 15 patients who survived 60 months or more after surgery. This analysis of the different survival groups revealed 15 differentially methylated genes. *RTEL1*, the regulator of telomere elongation helicase 1, seems to be the most functionally interesting one of these. This is an ATP-dependent DNA helicase required to suppress inappropriate homologous recombination, thereby playing a central role in DNA repair and in the maintenance of genomic stability. *RTEL1* was found to be hypermethylated in our poor survival group.

Comparing non-smokers (n = 3, 6.4%) with smokers (n = 44, 93.6%), four differentially methylated CpG-sites related to three genes, hypermethylated in the non-smokers group were found: *CXorf38, MTHFD2* and *TLL2*. Missing statistically significant difference in methylation levels between the different levels of tobacco smoking could indicate a poor reliability of the pack-years’ data obtained. *MTHFD2* has been shown to have a higher expression in smokers, favouring rapid cell growth. [33]. *TLL2* is a zinc-dependent metalloprotease. Expression of metalloproteinases is required for cell transformation, and this correlates with tumor progression [34].

By comparing genome-wide changes in the DNA methylation of normal and lung cancer cells, we were able to gain insight into the complexity of the methylation program required for cells to become fully malignant. As a result, we found a panel of genes that distinguish NSCLC cells from adjacent normal lung cells and also squamous cell carcinoma from adenocarcinoma. The analysis revealed a set of differentially methylated CpG sites that appear to regulate gene expression and another set that had affected the survival of the patients. These newly-identified methylation markers are candidates for the further molecular screening of NSCLC. In order to confirm these markers of NSCLC carcinogenesis, additional studies and validations are needed. From the results of our work and from previous findings, we can assume that altered DNA methylation is an early event in NSCLC.

## Materials and Methods

### Ethics Statement

The Ethics Committee of Human Studies, University of Tartu, has approved the study and a written consent was obtained from the study subjects.

### Samples

We analyzed Union for International Cancer Control (UICC) stage I NSCLC samples [35] from 48 patients and macroscopically cancer-free “normal” lung control samples from 18 patients. All the specimens had been isolated during lung surgery at Tartu University Hospital, Estonia. The patients with adenocarcinoma (n = 6, 12.5%) and its subtype bronchioloalveolar carcinoma (n = 10, 20.8%) were analyzed as one group (n = 16, 33.3%). The remaining 32 (66.7%) of the analyzed patients had squamous cell carcinoma. The age range in the study group was 41–80 years (mean age in males n = 40, 66.2 years and in females n = 8, 65.5 years) (Table S3). The patients did not undergo any preoperative chemo- or radiotherapy.

At surgery, tissue specimens of appropriate size (1–2 cm^3^) were cut from tumorous and morphologically tumor-free lung tissue. One half of each sample was fixed in formalin and used for pathological examination. The other half of each specimen was snap frozen and stored at −80°C until use. Control samples were obtained at a site distant from the removed tumor and confirmed to be tumor-free by the same pathologist.

### DNA Extraction and Bisulfite Modification

DNA was extracted from 50 mg of tumor and matching tumor-free lung tissue with the Dneasy® Blood & Tissue kit (Qiagen GmbH., Hilden, Germany) and with the Nucleospin® Tissue kit (Macherey-Nagel GmbH., Düren, Germany). DNA yield and purity were determined using the NanoDrop® ND1000 spectrophotometer (Thermo Fisher Scientific Inc., Waltham, MA). From each sample, 500 ng of genomic DNA was bisulfite modified using the EZ DNA Methylation™ Kit (Zymo Research, Orange, CA) according to the manufacturer’s recommendations.

### Methylation Validation by Sanger Sequencing

For methylation chip validation 11 genes were chosen, five of these were genes from survival analysis and the remaining six were genes that distinguished between cancer and normal tissue (Primers used in study showed in Table S4). Primers for bisulfite-treated DNA PCR were designed using MethPrimer [36]. A 20 µl PCR was carried out in 80 mM Tris-HCl (pH 9,4–9,5), 20 mM (NH4)2SO4, 0,02% Tween-20 PCR buffer, 3 mM MgCl2, 1X Betaine, 0.25 mM dNTP mix, 2 units of Hot-start Taq polymerase, 50 pmol of the forward primer, 50 pmol of the reverse primer, and 20 ng of bisulfite-treated genomic DNA. PCR cycling conditions were 95°C 15 min for enzyme activation, 95°C 30 sec, 62°C 45 sec, 72°C 120 sec for 17 cycles, touchdown by −0.5°C for every cycle and 95°C 30 sec, 52°C 30 sec, 72°C 120 sec for 21 cycles. Sequencing was done as a service by the Core Facility of Estonian Biocenter. We analyzed sequencing traces with Mutation Surveyor software (Softgenetics, State College, PA, USA) and R statistical computing software (http://www.r-project.org/).

### RNA Extraction and Gene Expression Analysis

Detailed description of the RNA extraction and gene expression analysis process is given in the recent paper [14].

10 genes and eight sample pairs (tumor and adjacent normal sample) were used to validate the microarray data. For quantitative RT-PCR (qPCR), cDNAs were synthesized from 700 ng of total RNA using the First Strand cDNA Synthesis kit (Fermentas, Vilnius, Lithuania) and oligo dT primers according to the manufacturer’s protocol. Triplicate qPCR reactions were performed in 384-well plates using SYBR Green ROX mix (ABGene, Epsom, UK or Fermentas, Vilnius, Lithuania) and ABI 7900HT Sequence Detection System (Applied Biosystems, Foster City, CA). Data were analyzed using the SDS 2.2.2 (Applied Biosystems) and R statistical computing software (http://www.r-project.org/).

The geometric mean expression of two reference genes (*ESD* and *S18RNA*) was used as a reference. Expression fold change between normal and tumor sample were calculated using 2^-ΔΔCt^ method. Pearson correlation analyses were used to assess the accordance between fold changes identified by qRT-PCR and array experiments.

In addition, qRT-PCR was used to determine the *TP73* expression level. Primers used for the *TP73* qPCR amplifications are listed in Table S5.

### DNA Methylation Analysis

Methylation analysis was performed using Infinium® HumanMethylation27 RevB BeadChips (Illumina Inc.). The assay covers 27,578 CpGs in 14,495 genes located predominantly in CpG islands within proximal promoter regions, between 1.5 kb upstream and 1 kb downstream of the transcription start sites (TSS). A CpG island in this assay is defined as a nucleotide sequence of (1) 200 bp or greater in length, (2) 50% or greater in GC-percent, and (3) 0.60 or greater in the ratio of observed CpG sites over expected CpG sites in that region [10]. The HumanMethylation27 beadchips also cover CpG sites in the regulatory regions of 1,000 well-known cancer genes, 150 differentially methylated genes in various cancers and 110 miRNA genes. The chips were processed according to the manufacturer’s standard protocols.

We performed cluster analysis of the methylation profiles by using the Limma program of Bioconductor package in R statistical computing software (www.bioconductor.org). Prior to analysis we quantile normalized the methylation data to eliminate systematic differences between the chips. The analysis was performed using t-tests with an empirical Bayes’ correction from the Bioconductor Limma package [37]. The differentially methylated genes were clustered hierarchically and visualized using a heatmap.

All the methylation differences (differences between the Beta-values representing the calculated level of methylation from 0 to 1, alternatively 0% to 100% for each analyzed CpG) were identified using a false discovery rate (FDR) corrected p<0.05 and ≥0.136 mean methylation level difference that was previously shown to detect differences with at least 95% confidence [38]. For Kaplan-Meier survival analysis, we divided the Beta values into low (0–0.25), medium (0.25–0.75) and high (0.75–1) methylation group and performed a log-rank test to assess the difference in survival between the groups. We corrected these p-values using FDR and used 0.05 as the significance level.

### Methylation Related to Gene Expression Analysis

Within the available data, we calculated Pearson’s correlations between the CpG site methylation levels and their respective gene expression levels (Table 2, Table S1 and S2).

**Table 2:** Statistically significant Pearson’s correlations between differentially methylated CpG sites and gene expression values across 48 lung cancer samples and the control samples with available gene expression data.

Symbol	Gene ID	Pearson R	Permuted p-value*
AGER	177	−0.8095317	0.0143
BRDT	676	−0.7664438	0.0477
CALML5	51806	−0.7729556	0.0419
ELAVL4	1996	−0.8363362	0.0027
GSTT1	2952	−0.7698949	0.0450
MAGEC1	9947	−0.7886890	0.0278
MB	4151	−0.7765738	0.0390
NR0B2	8431	−0.8705916	0.0002
P53AIP1	63970	−0.7665071	0.0477
PNLDC1	154197	−0.8677312	0.0002
PPP1R14D	54866	−0.8137714	0.0121

P-values were computed by permuting individuals and recalculating the median gene expression levels 1,000 times. All CpGs and genes represented by both methylation and gene expression arrays were included in permutations.

* - Permuted p-value - p-value computed by permuting individuals and recalculating the median gene expression levels 1,000 times.

We computed the permutation-based p-values by permuting individuals and recalculating the median gene expression levels 10,000 times. All CpGs and genes represented by both the methylation and gene expression arrays were included in permutations. Three genes (AGER, NR0B2, PPP1R14D) yielded two significant correlations; only the strongest correlations are shown (Table 2).

## Supporting Information

Figure S1 **Differential DNA methylation between NSCLC and normal lung samples.** DNA methylation profiles for 48 NSCLC samples and 18 normal lung samples are shown. We detected 496 CpGs hyper- and 373 CpGs hypomethylated in NSCLC. Methylation values (Beta-values) are represented as row Z-scores for each gene. Heatmap was generated with unsupervised 2D hierarchical cluster analysis. Red indicates high methylation and blue indicates low methylation relative to the row mean.
(PDF)

Figure S2 **Differential DNA methylation between squamous cell carcinoma and adenocarcinoma.** DNA methylation profiles for 16 adenocarcinoma (AC) samples and 32 squamous cell carcinoma (SCC) samples are shown. We detected 263 hypermethylated CpG sites and 513 hypomethylated CpG sites comparing SCC to AC. Methylation values are represented as row Z-scores for each gene. Red indicates high methylation and blue indicates low methylation relative to the row mean.
(PDF)

Figure S3 **Scatterplots showing the correlation between beta values determined by Illumina methylation arrays and Sanger bisulfite sequencing.** Shown are the best-fitting line, Pearson’s correlation coefficient and p-value of correlation tests. The mean correlation between two methods was 83% (range 48.9%; 97.4%).
(DOC)

Figure S4 **Scatterplots showing the correlation between Illumina microarray and qRT-PCR measurements.** On the x-axis is quantile-normalized and logarithmic fold change (log2TU-log2N) on the microarray for eight sample pairs. On the y-axis is ΔΔCt value for same sample pairs. Shown are the best-fitting line, Pearson’s correlation coefficient and correlation test p-value.
(TIFF)

Figure S5 **TNF network.** The *in silico* functional and interaction analysis of differently methylated genes was performed using the Ingenuity Pathway Analysis (IPA) software. The most prominently represented gene network was related to tumor necrosis factor. The genes that were hypermethylated in NSCLC are shown with a red background, and the genes that were hypomethylated in NSCLC are shown with a green background. The depicted interactions are mostly indirect.
(TIF)

Figure S6 **Boxplots of differentially methylated CpGs in different survival groups.** The 18 CpGs in 15 genes had statistically different methylation (p-value <0.05, Beta-value ≤0.136) between patients with 1 to 24 months survival (n = 12) vs patients with 60 months and longer survival (n = 15). Methylation values are also shown for patients with 25–59 months survival and for normal lung tissue.
(PDF)

Table S1 **Differentially methylated CpGs and genes in stage I NSCLC compared to cancer-free lung control samples.** Mean Beta represents the methylated signals divided by the sum of methylated and unmethylated signals for each analyzed CpG. Beta-diff. was calculated by subtracting the Mean Beta value of a cancer-free lung from the Mean Beta value of stage I NSCLC samples.
(XLS)

Table S2 **Differentially methylated CpGs and genes in squamous cell carcinoma (SCC) compared to adenocarcinoma (AC) samples.** Mean Beta represents the methylated signals divided by the sum of methylated and unmethylated signals for each analyzed CpG. Beta-diff. was calculated by subtracting the Mean Beta value of AC from the Mean Beta value of SCC.
(XLS)

Table S3 **A detailed overview of the patient cohort involved in our study.**
(DOC)

Table S4 **PCR primers used in methylation validation with Sanger sequencing.**
(DOC)

Table S5 **Quantitative real-time PCR primers used for TP73 gene isoform analysis.**
(DOC)
