# Correction: Methylation Markers of Early-Stage Non-Small Cell Lung Cancer

**DOI:** 10.1371/journal.pone.0170833

**Published:** 2017-01-20

**Authors:** Kaie Lokk, Tõnu Vooder, Raivo Kolde, Kristjan Välk, Urmo Võsa, Retlav Roosipuu, Lili Milani, Krista Fischer, Marina Koltsina, Egon Urgard, Tarmo Annilo, Andres Metspalu, Neeme Tõnisson

The incorrect file for S3 Fig is included in the Supporting Information. Please see the correct S3 Fig here.

## Supporting Information

S3 Fig Scatterplots showing the correlation between beta values determined by Illumina methylation arrays and Sanger bisulfite sequencing. Shown are the best-fitting line, Pearson’s correlation coefficient and p-value of correlation tests. The mean correlation between two methods was 83% (range 48.9%; 97.4%).
(TIFF)
