# Supplementary figures and images for: Methylation Markers of Early-Stage Non-Small Cell Lung Cancer
Source: PLoS One. 2012 Jun 29;7(6):e39813. doi: 10.1371/journal.pone.0039813 (PMC3387223; doi:10.1371/journal.pone.0039813)

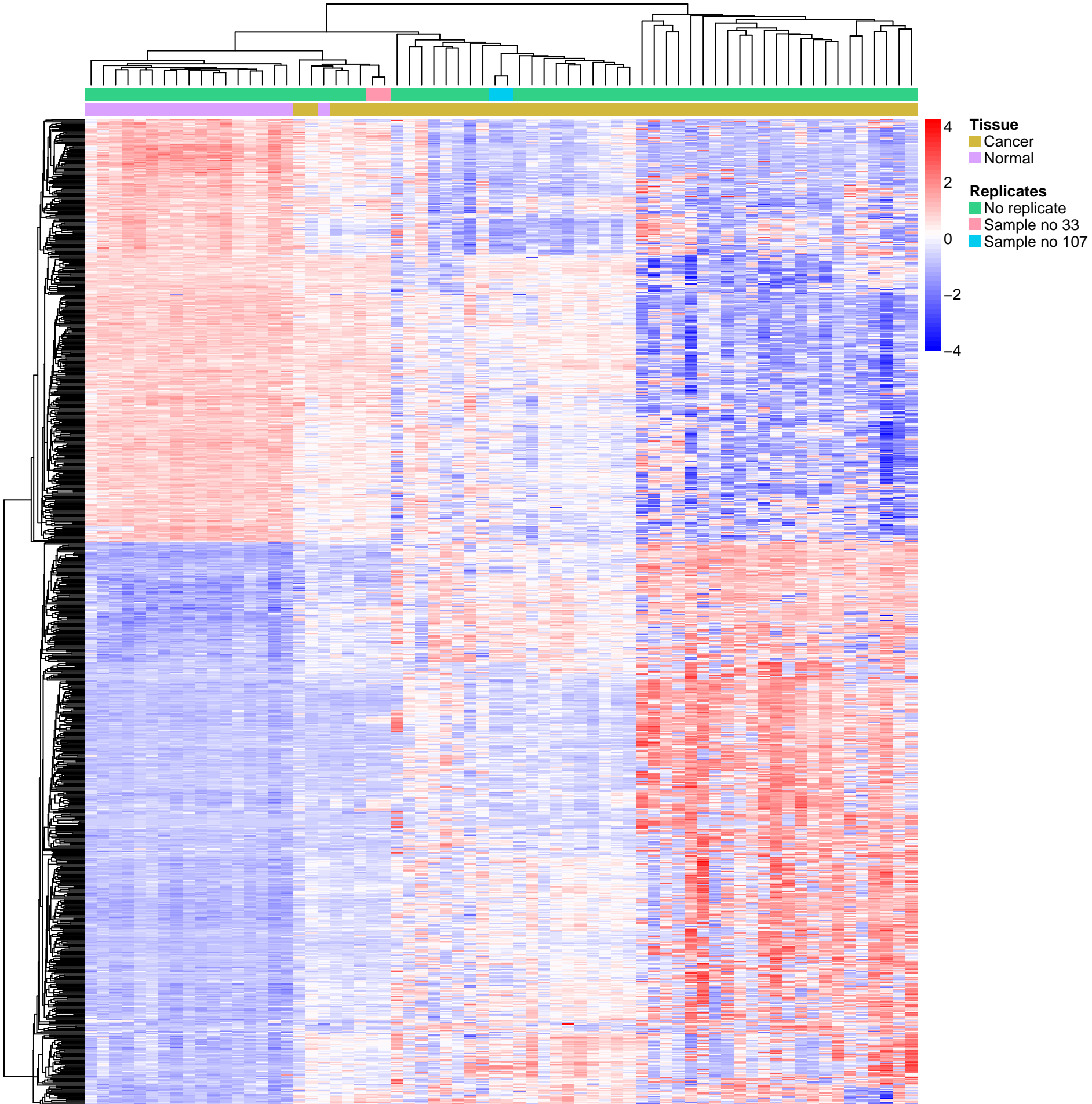

Supplement: Figure S1 — Differential DNA methylation between NSCLC and normal lung samples. DNA methylation profiles for 48 NSCLC samples and 18 normal lung samples are shown. We detected 496 CpGs hyper- and 373 CpGs hypomethylated in NSCLC. Methylation values (Beta-values) are represented as row Z-scores for each gene. Heatmap was generated with unsupervised 2D hierarchical cluster analysis. Red indicates high methylation and blue indicates low methylation relative to the row mean. (PDF) [file pone.0039813.s001.pdf]

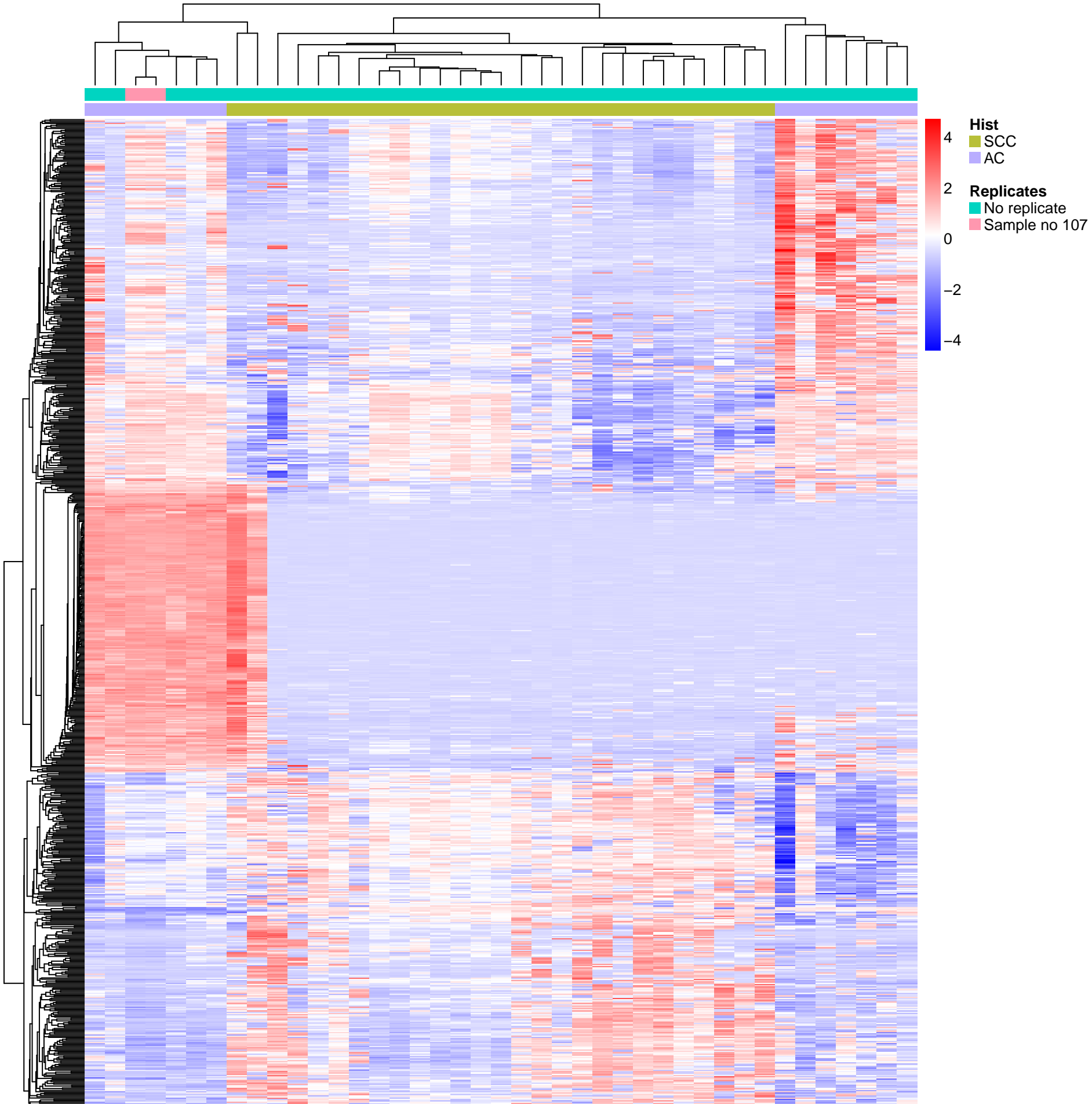

Supplement: Figure S2 — Differential DNA methylation between squamous cell carcinoma and adenocarcinoma. DNA methylation profiles for 16 adenocarcinoma (AC) samples and 32 squamous cell carcinoma (SCC) samples are shown. We detected 263 hypermethylated CpG sites and 513 hypomethylated CpG sites comparing SCC to AC. Methylation values are represented as row Z-scores for each gene. Red indicates high methylation and blue indicates low methylation relative to the row mean. (PDF) [file pone.0039813.s002.pdf]

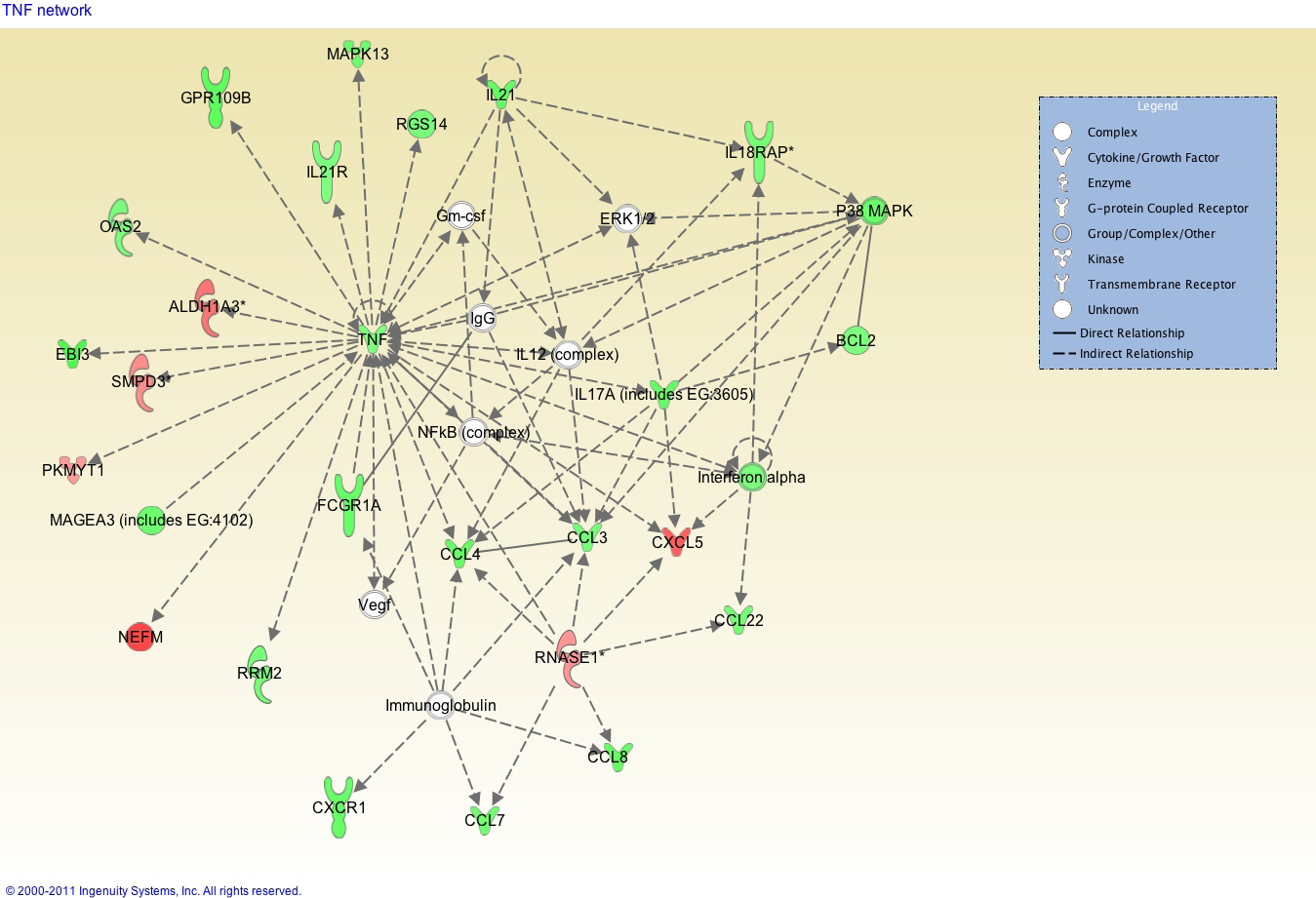

Supplement: Figure S5 — TNF network. The in silico functional and interaction analysis of differently methylated genes was performed using the Ingenuity Pathway Analysis (IPA) software. The most prominently represented gene network was related to tumor necrosis factor. The genes that were hypermethylated in NSCLC are shown with a red background, and the genes that were hypomethylated in NSCLC are shown with a green background. The depicted interactions are mostly indirect. (TIF) [file pone.0039813.s005.tif]
