# Supplementary material for: Methylation Markers of Early-Stage Non-Small Cell Lung Cancer
Source: PLoS One. 2012 Jun 29;7(6):e39813. doi: 10.1371/journal.pone.0039813 (PMC3387223; doi:10.1371/journal.pone.0039813)
Supplement: Figure S6 — Boxplots of differentially methylated CpGs in different survival groups. The 18 CpGs in 15 genes had statistically different methylation (p-value <0.05, Beta-value ≤0.136) between patients with 1 to 24 months survival (n = 12) vs patients with 60 months and longer survival (n = 15). Methylation values are also shown for patients with 25–59 months survival and for normal lung tissue. (PDF) [file pone.0039813.s006.pdf]

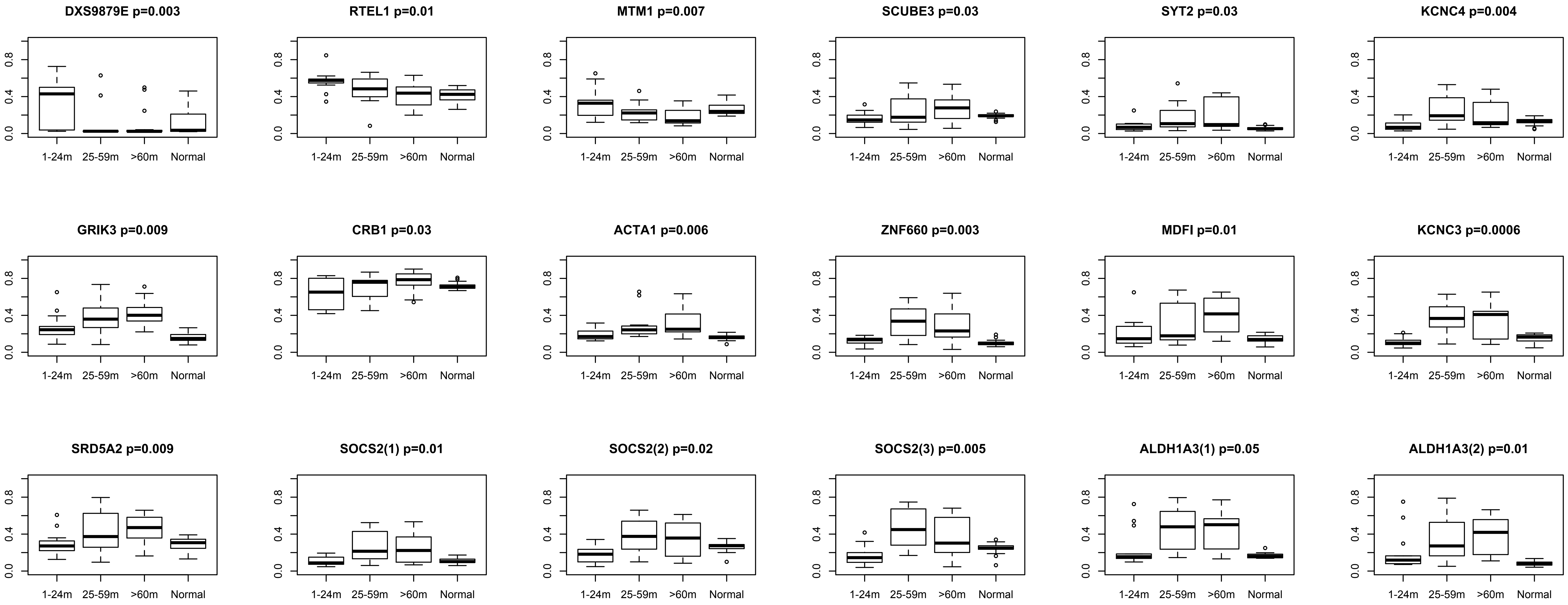
DXS9879E p=0.003
RTEL1 p=0.01
MTM1 p=0.007
SCUBE3 p=0.03
SYT2 p=0.03
KCNC4 p=0.004
GRIK3 p=0.009
CRB1 p=0.03
ACTA1 p=0.006
ZNF660 p=0.003
MDFI p=0.01
KCNC3 p=0.0006
SRD5A2 p=0.009
SOCS2(1) p=0.01
SOCS2(2) p=0.02
SOCS2(3) p=0.005
ALDH1A3(1) p=0.05
ALDH1A3(2) p=0.01
0.0
0.4
0.8
1-24m
25-59m
>60m
Normal
