# Supplementary material for: Methylation Markers of Early-Stage Non-Small Cell Lung Cancer
Source: PLoS One. 2012 Jun 29;7(6):e39813. doi: 10.1371/journal.pone.0039813 (PMC3387223; doi:10.1371/journal.pone.0039813)
Supplement: Table S3 — A detailed overview of the patient cohort involved in our study. (DOC) [file pone.0039813.s009.doc]

|  | **Number of patients** | % |
| --- | --- | --- |
| Patients involved | 48 |  |
| Age range | 41 - 80 |  |
| Male | 40 | 83,33% |
| Range | 44 - 80 |  |
| Average | 66,23 |  |
| Median | 67,5 |  |
| Female | 8 | 16,67% |
| Range | 41 - 79 |  |
| Average | 65,5 |  |
| Median | 67 |  |
|  |  |  |
| Histology |  |  |
| Adenocarcinoma | 6 | 12,5% |
| BA | 10 | 20,83% |
| Squamocellular | 32 | 66,67% |
|  |  |  |
| Lymphnode negative | 48 | 100% |
|  |  |  |
| Differentiation |  |  |
| Moderate | 46 | 95,83% |
| poor | 2 | 4,17% |
|  |  |  |
| Smoking history |  |  |
| yes | 46 | 95,83% |
| no | 2 | 4,17% |
| Family history of cancer | 4 | 8,3% |
| Stage |  |  |
| Ia | 13 | 27,1% |
| Ib | 35 | 72,9% |
| T1 | 13 | 27,1% |
| T2 | 35 | 72,9% |
| Tumour size |  |  |
| <3,0 cm | 20 | 41,7% |
| >3,0 cm | 28 | 58,3% |
| Surgical procedure |  |  |
| Wedge resection | 2 | 4,17% |
| Lobectomy | 38 | 79,17% |
| Bilobectomy | 2 | 4,17% |
| Pneumonectomy | 6 | 12,5% |
