# Supplementary material for: Methylation Markers of Early-Stage Non-Small Cell Lung Cancer
Source: PLoS One. 2012 Jun 29;7(6):e39813. doi: 10.1371/journal.pone.0039813 (PMC3387223; doi:10.1371/journal.pone.0039813)
Supplement: Table S4 — PCR primers used in methylation validation with Sanger sequencing. (DOC) [file pone.0039813.s010.doc]

#### Table S4

PCR primers used in methylation validation with Sanger sequencing

| **Gene** | **Product size (bp)** | **Left Primer 5'-3'** | **Right Primer 5'-3'** |
| --- | --- | --- | --- |
| UGT1A7 | 382 | TTGTTAATTTATTTTTGGTATGATTA | AATATCTCAACAAAAACTACTCATATATTA |
| PIK3R5 | 270 | AAAGAGGGATAAAATTTTAATTTGG | CACATAACAAACAATCAATAAACCC |
| GRIK3 | 262 | TTTTTTTTAGGGTTTTTATTTTG | TAACCACCAAAAACTCCTCC |
| CLEC11A | 267 | TGGGTTATAGTAGAAGGAAAGAGTAGG | ATACAAATCACTAACCCCCAAATT |
| C20orf185 | 263 | TTTTTGGTTAGTGATATGTTGTTGAAT | AACATTACCTCCCTAAAAAAACCTC |
| AGER | 207 | AAATAGTGTAAGGTTTTTGGGAAAG | AATAAATACTCAAAACATCACAACCC |
| AQP1 | 279 | AGTTTTTAGTTAGGGAGGAGGTGAT | ATTCTACCCTAAACTTCAAATACCC |
| ADA | 246 | TGATTTGATTTTTTTGGGTTTTAGT | CATCCACAAACCTAAACTCCTTAAC |
| MAGEA6 | 290 | GGTAGAATTTAGTTTTATTTTTGTT | CAACCTAAAAATCTTCCCCTAC |
| TRIM15 | 232 | GGTTATTATGGGTAGATGTGGTGAG | AACAAAAACTTCCTATCAAATTAAACAA |
| MB | 408 | TTTAGATGGAAGGGTAGAGGTGTAG | ACCCTTTCTTTTACAAAAAACACTTT |
