# Supplementary material for: Methylation Markers of Early-Stage Non-Small Cell Lung Cancer
Source: PLoS One. 2012 Jun 29;7(6):e39813. doi: 10.1371/journal.pone.0039813 (PMC3387223; doi:10.1371/journal.pone.0039813)
Supplement: Table S5 — Quantitative real-time PCR primers used for TP73 gene isoform analysis. (DOC) [file pone.0039813.s011.doc]

# Supplemental Data Table 2 - qPCR primers used for TP73 gene isoform analysis

| Primer name | Sequence |
| --- | --- |
| GAPDH-L | 3’-GGTGGTCTCCTCTGACTTC-5’ |
| GAPDH-R | 3’-CTCTTCCTCTTGTGCTCTTG-5’ |
| TP73long_L | 3’-GGAACCAGACAGCACCTACTT-5’ |
| TP73long_R | 3’-CTCAGCAGATTGAACTGGGC-5’ |
| TP73short_L | 3’-CCACGGCCCAGTTCAATC-5’ |
| TP73short_R | 3’-GTTGTGCGTAGGGCGAGT-5’ |
